# Pathological alpha-synuclein propagates through neural networks

**DOI:** 10.1186/s40478-014-0088-8

**Published:** 2014-08-06

**Authors:** Masami Masuda-Suzukake, Takashi Nonaka, Masato Hosokawa, Maki Kubo, Aki Shimozawa, Haruhiko Akiyama, Masato Hasegawa

**Affiliations:** Department of Neuropathology and Cell Biology, Tokyo Metropolitan Institute of Medical Science, 2-1-6 Kamikitazawa, Setagaya-ku, Tokyo 156-0057 Japan; Dementia Research Project, Tokyo Metropolitan Institute of Medical Science, Setagaya-ku, Tokyo Japan

**Keywords:** α-Synuclein, Lewy bodies, Propagation, Prion

## Abstract

**Background:**

α-Synuclein is the major component of filamentous inclusions that constitute the defining characteristic of Parkinson’s disease, dementia with Lewy bodies and multiple system atrophy, so-called α-synucleinopathies. Recent studies revealed that intracerebral injection of recombinant α-synuclein fibrils into wild-type mouse brains induced prion-like propagation of hyperphosphorylated α-synuclein pathology. However, the propagation mechanisms of α-synuclein have not been fully elucidated.

**Results:**

In this study, in order to establish where and how α-synuclein pathology propagates, we injected recombinant mouse α-synuclein fibrils into three different brain areas (substantia nigra, striatum, and entorhinal cortex) of wild-type mice and compared the resulting distributions of α-synuclein pathology at 1 month after injection. Distinct patterns of pathology were observed in mice injected at the different sites. Within one month after injection, the pathology had spread to neurons in areas far from the injection sites, especially areas with direct neural connections to the injection sites. Surprisingly, phosphorylated tau and TDP-43 pathologies were also observed in mice injected with α-synuclein fibrils into striatum and entorhinal cortex at one month after injection. Phosphorylated tau and TDP-43 were accumulated in dot-like inclusions, but these were rarely colocalized with α-synuclein pathology. It seems that accumulation of α-synuclein has a synergistic effect on tau and TDP-43 aggregation. Additionally, intracerebral injection with sarkosyl-insoluble fraction prepared from wild-type mice injected synthetic α-synuclein fibrils can also induce phosphorylated α-synuclein pathology in wild-type mice.

**Conclusions:**

Our data indicate that α-synuclein aggregation spread by prion-like mechanisms through neural networks in mouse brains.

**Electronic supplementary material:**

The online version of this article (doi:10.1186/s40478-014-0088-8) contains supplementary material, which is available to authorized users.

## Introduction

Parkinson’s disease (PD) and dementia with Lewy bodies (DLB) are progressive neurodegenerative diseases characterized by appearance of Lewy bodies (LBs) and Lewy neurites (LNs) [1]. α-Synuclein (αsyn) is the major component of LBs and LNs, and is deposited in a hyperphosphorylated form in β-sheet-rich amyloid fibrils [2–5]. Five missense mutations in the αsyn gene and occurrence of gene multiplication have been identified in the familial forms of PD and DLB [6–13]. Moreover, it was reported that the mutations affect amyloid fibril formation *in vitro*, either accelerating fibril formation [14–16] or resulting in formation of fibrils that are more fragile and easy to propagate than wild-type (WT) fibrils [17]. These results clearly indicate that abnormalities of αsyn can induce PD and DLB. Distribution of αsyn pathology in brains with sporadic PD occurs from olfactory bulb and/or brainstem, and spreads to other brain regions concomitantly with progression of disease symptoms [18,19]. Thus, spread of αsyn pathology in the brain can be regarded as the underlying mechanism of progression of these diseases. Recently intracerebral injection of synthetic αsyn fibrils and/or insoluble αsyn from diseased brain was shown to induce αsyn pathology that propagated throughout the brain in a prion-like manner in WT mouse [20,21], αsyn transgenic mouse [22–24] and monkey [25]. However, the mechanisms through which exogenous abnormal fibrils enter neurons and through which insoluble αsyn is transported to other neurons remain unknown.

To investigate where αsyn pathologies develop and how they propagate, we injected recombinant αsyn fibrils into substantia nigra, striatum, or entorhinal cortex of WT mice, and compared the spreading patterns and distribution of phosphorylated αsyn pathologies at 1 month after intracerebral injections. Our results clearly suggest that propagation of pathological αsyn occurred along neural circuits and involved trans-synaptic transport. We also showed that αsyn pathology induced tau and TDP-43 accumulation in WT mice, similar to that seen in DLB brains. This mouse model should be useful for elucidating mechanisms of disease progression of synucleinopathy and also for development of novel disease-modifying drugs.

## Materials and methods

### Antibodies

Antibodies used in this study are summarized in Additional file 1: Table S1. 1175 polyclonal antibody was raised against an αsyn peptide phosphorylated at serine 129 [21,26]. Anti-phosphorylated αsyn mouse monoclonal antibody, #64 [5] and anti-human αsyn specific mouse monoclonal antibody, LB509 [27] were kindly provided from Dr. Iwatsubo. Rabbit polyclonal pS396 antibody (Calbiochem) is specific for phosphorylated tau at serine 396; biotin-AT8 (Thermo Scientific) is specific for phosphorylated tau at serine 202/threonine 205; anti-mouse αsyn rabbit monoclonal antibody (Cell Signaling Technology) is specific for mouse αsyn. Rabbit polyclonal pTDP-43 antibody is specific for phosphorylated at serine 409/410 [28].

### Preparation of recombinant αsyn monomer and fibrils

Mouse αsyn cDNA in bacterial expression plasmid pRK172 was used. αSyn were expressed in *Escherichia coli* BL21 (DE3) cells and purified using boiling, Q-sepharose ion exchange chromatography and ammonium sulfate precipitation. Purified αsyn protein was dialyzed against 30 mM Tris–HCl, pH 7.5, and cleared using ultracentrifugation at 113,000 g for 20 min. Protein concentration was determined by reverse phase HPLC. Proteins were loaded on an Aquapore RP-300 column (PerkinElmer Brownlee) equilibrated in 0.09% trifluoroacetic acid with linear gradient of acetonitrile 0 to 50% at a flow rate of 1 ml/min [21]. Purified mouse αsyn monomer (7 mg/ml) in 30 mM Tris–HCl, pH 7.5, containing 0.1% NaN_3_ was incubated at 37°C in a shaking incubator at 200 rpm for 72 h. αSyn fibrils were pelleted by spinning at 113,000 g for 20 min and suspended in PBS. αSyn fibrils were sonicated with a ultrasonic homogenizer (VP-5S, TAITEC) before use. To determine the concentration, fibrils were dissolved in 8 M guanidine hydrochloride and analyzed by RP-HPLC as described above.

### Mice

C57BL/6 J mice, used as WT mice, were purchased from CLEA Japan, Inc. αSyn (SNCA) knockout mice [29] were purchased from the Jackson Laboratory.

### Stereotaxic surgery

Four- to six-month-old mice anesthetized with 50 mg/kg pentobarbital sodium were unilaterally injected with 10 μg of recombinant mouse αsyn fibrils into substantia nigra (SN, n = 6) (A-P: −3.0 mm; M-L: −1.3 mm; D-V: −4.7 mm from the bregma and dura) [21], striatum (Str, n = 6) (A-P: 0.2 mm; M-L: −2.0 mm; D-V: −2.6 mm) [20], or entorhinal cortex (EC, n = 6) (A-P: −3.1 mm; M-L: −4.0 mm; D-V: −2.7 mm). Mice were anesthetized with isoflurane and killed by decapitation. For immunohistochemistry (IHC, n = 3), brains were fixed in 10% formalin neutral buffer solution (Wako). For biochemical analysis (n = 3), brains were snap-frozen on dry ice and stored at −80°C. All experimental protocols were approved by the Animal Care and Use Committee of the Tokyo Metropolitan Institute of Medical Science.

### Peripheral injection of αsyn

For intraperitoneal injection, 2-month-old C57BL/6 J mice were injected intraperitoneally with 100 μg of mouse αsyn monomer or fibrils. At 6 months after injection, the pathology of mouse brains in both groups (n = 3 each) was tested by immunohistochemistry (IHC). For oral administration, 2- or 3-month-old C57BL/6 J mice were orally administrated with 400 μg of human αsyn monomer, human αsyn fibrils, mouse αsyn monomer or mouse αsyn fibrils every two weeks for 4 times. At 12 months post final administration, pathology in mouse brains (n = 3 each) was analyzed by IHC.

### Immunohistochemistry

Fixed brains were cut on a vibratome (Leica) at 50 μm thickness. For high-sensitivity detection, mouse brain sections were treated with formic acid for 30 min, washed, and boiled at 100°C for 30 min. The sections were then incubated with 0.5% H_2_O_2_ in methanol to inactivate endogenous peroxidases, blocked with 10% calf serum in PBS, and immunostained with appropriate antibodies. After incubation with the biotinylated-secondary antibody (Vector), labeling was detected using the ABC staining kit (Vector).

### Confocal microscopy

For double-label immunofluorescence to detect phosphorylated αsyn and tau, brain sections were incubated overnight at 4°C in a cocktail of #64 antibody and anti-pS396 antibody. The sections were washed and incubated in a cocktail of Alexa568-conjugated goat anti mouse IgG (Molecular Probes) and Alexa488-conjugated goat anti rabbit IgG (Molecular Probes). After further washing, sections were stained with TOPRO-3, coverslipped with Vectashield (Vector) and observed with a laser-scanning confocal fluorescence microscope (LSM5 PASCAL; Carl Zeiss).

### Biochemical analysis

Biochemical analysis of mouse brains (n = 3 per group) was conducted as described previously [21]. Briefly, brains were homogenized in 20 volumes (w/v) of buffer A (10 mM Tris–HCl, pH 7.4, 0.8 M NaCl, 1 mM EGTA and 10% sucrose), then spun at 100,000 g for 30 min at 4°C, and the supernatant was retained as buffer-soluble fraction. The pellet was homogenized in 20 volumes of buffer A containing 1% Triton X-100 and incubated for 30 min at 37°C. After centrifugation at 100,000 g, the Triton-insoluble pellet was further homogenized in buffer A containing 1% sarkosyl and incubated at 37°C for 30 min. Samples were spun at 100,000 g for 30 min. The sarkosyl-pellet was sonicated in 30 mM Tris–HCl, pH 7.4, and used for immunoblotting as sarkosyl-insoluble fraction. The samples were subjected to SDS-PAGE and proteins were electrotransferred onto a polyvinylidene difluoride membrane, probed with appropriate antibodies and detected as described previously [21].

### Behavioral tests

For behavioral tests, C57BL/6 J male mice were used. Mouse αsyn fibrils (10 μg) were injected into SN (n = 10), Str (n = 15), or EC (n = 14) of 3-month-old mice, and the same amount of mouse αsyn monomer was injected into SN (n = 9), Str (n = 8), or EC (n = 8) of control mice. At 3 months after injection, motor and cognitive activities were evaluated as described below.

### Rotarod test

Motor coordination and balance were measured in terms of performance on the rotarod. Mice were placed on 3-cm diameter rods and the speed of the rotation was increased from 0 to 40 rpm over 5 min. Latency to fall was recorded. Each mouse was tested three times and the average was used. Statistical analyses were performed using Student’s *t*-test.

### Wire hang test

Neuromuscular abnormalities were tested with the wire hang test. The mouse was placed on a wire cage lid, which was waved gently so that the mouse gripped the wire and then inverted. Latency to fall was recorded with a 300-sec cut-off time. The test was conducted three times and statistical analyses were performed using Student’s *t*-test.

### Y-maze test

The Y-maze apparatus (Muromachi kikai) consisted of three arms (40 cm × 3 cm) made of grey plastic joined in the middle to form a Y shape. Mice were placed into one of the arms of the maze and allowed to freely explore the three arms for an 8-min session. Alternation between arms was recorded. The Y-maze test was conducted twice. Statistical analyses were performed using Student’s *t*-test.

### Transmission experiments

Recombinant human αsyn fibrils (10 μg) were injected into SN of 4-month-old WT mice (n = 4). At 9 months after injection, sarkosyl-insoluble pellets were prepared from the whole brains as described above, collected in one tube, and stored at −80°C until use. Sarkosyl-insoluble pellets were suspended in 100 μl PBS and sonicated for 30 seconds (TAITEC, VP-5S), and 5-μl aliquots were injected into Str of 4-month-old WT mice (n = 10). At 3 months post injection, pathology was analyzed by IHC.

## Results

**Figure 1:**
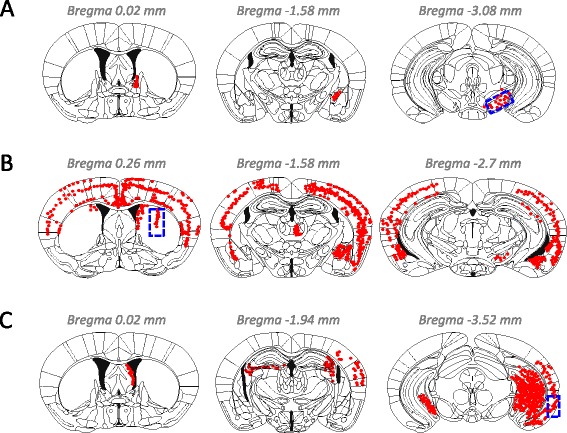
**Distribution of phosphorylated αsyn pathology in αsyn fibril-injected mice at 1 month after injection. (A)** Injection into SN induced αsyn pathology mainly in SN (3.08 mm posterior to bregma), amygdala (1.58 mm posterior to bregma) and stria terminalis (0.02 mm anterior to bregma). **(B)** Injection into Str induced severe αsyn pathology throughout the brain, including Str (0.26 mm anterior to bregma), amygdala (1.58 mm posterior to bregma), SN (2.70 mm posterior to bregma) and a wide range of cortex. **(C)** Injection into EC induced αsyn pathology that was concentrated in EC (3.52 mm posterior to bregma), dentate gyrus (3.52 mm posterior to bregma), CA3 (3.52 mm posterior to bregma), fimbria (1.94 mm posterior to bregma), and septal nucleus (0.02 mm anterior to bregma). Blue-dashed box and red dots indicate the injection site and psyn pathology, respectively.

**Figure 2:**
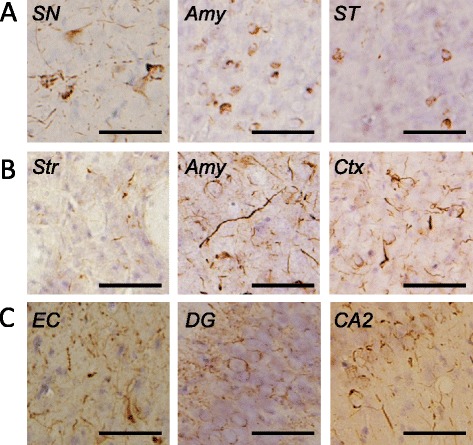
**Staining of WT mouse brains injected with αsyn fibrils at 1 month after injection by using 1175 antibody. (A)** Psyn pathology in mice injected into SN. **(B)** Psyn pathology in mice injected into Str. **(C)** Psyn pathology in mice injected into EC. SN: substantia nigra, Amy: amygdala, ST: stria terminalis, Str: striatum, Ctx: cortex, EC: entorhinal cortex, DG: dentate gyrus. Scale bar represents 50 μm.

We investigated the spread of αsyn pathology in brains of mice after unilateral injection of recombinant mouse αsyn fibrils into SN, Str, or EC. We confirmed the purity of recombinant αsyn monomer and fibrils used in this study; they didn’t contain any contaminants (Additional file 1: Figure S1). Using highly sensitive immunohistochemistry (IHC) with anti-phosphorylated αsyn (psyn) antibody 1175, we evaluated αsyn pathology in the brains at 1 month after injection. The distribution of αsyn pathology observed in these mice is illustrated in Figure 1. In mice injected into SN (Figure 1A), abnormal psyn pathology was restricted mainly to SN (3.08 mm posterior to bregma), amygdala (1.58 mm posterior to bregma), and stria terminalis (0.02 mm anterior to bregma) of the hemisphere on the injection side. In these mice, psyn was accumulated in neurites and soma (Figure 2A). In mice injected into Str, psyn pathology was widely distributed bilaterally throughout the brain, including Str (0.26 mm anterior to bregma), amygdala (1.58 mm posterior to bregma), SN (2.70 mm posterior to bregma) and cortex (Figure 1B). Psyn pathology was accumulated mainly in neurites, and partly in soma (Figure 2B). Injection of αsyn fibrils into EC induced severe psyn pathology in EC (3.52 mm posterior to bregma), dentate gyrus (3.52 mm posterior to bregma), hippocampal CA3 region (1.94 and 3.52 mm posterior to bregma), fimbria (1.94 mm posterior to bregma), and septal nuclei (0.02 mm anterior to bregma) on the injection side, as well as moderate psyn pathology in hippocampus on the contralateral side (Figure 1C). Psyn pathology was mainly observed in neurites and perinuclear regions (Figure 2C). No such psyn accumulation was detected in αsyn KO mice injected with αsyn fibrils into Str (Additional file 1: Figure S2A). Thus, there are major differences among these mice in the development and spread of αsyn pathologies, demonstrating that the propagation pattern depends upon the injection site.

**Figure 3:**
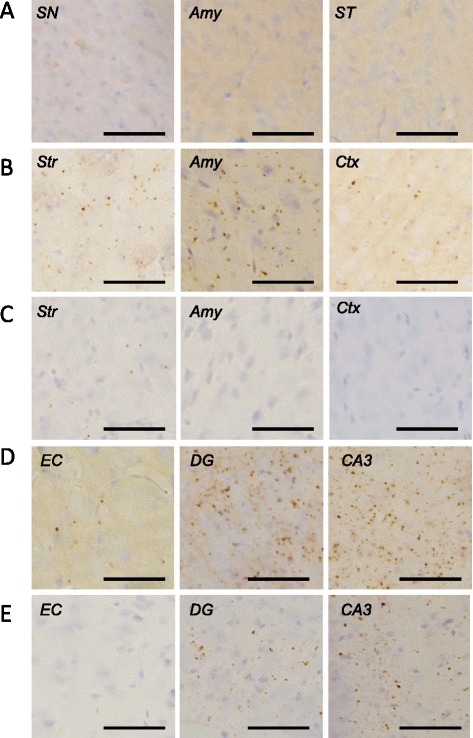
**Induction of phosphorylated tau inclusions in WT mice injected with αsyn fibrils at 1 month after injection. (A)** Phosphorylated tau was not accumulated in mice injected into SN, based on staining with anti-pS396 antibody. **(B-C)** Dot-like tau inclusions were observed in mice injected into Str by using anti-pS396 antibody **(B)** and AT8 **(C) (D-E)**. Dot-like tau inclusions were also detected in mice injected into EC by using anti-pS396 antibody **(D)** and AT8 **(E)**. SN: substantia nigra, Amy: amygdala, ST: stria terminalis, Str: striatum, Ctx: cortex, EC: entorhinal cortex, DG: dentate gyrus. Scale bar represents 50 μm.

**Figure 4:**
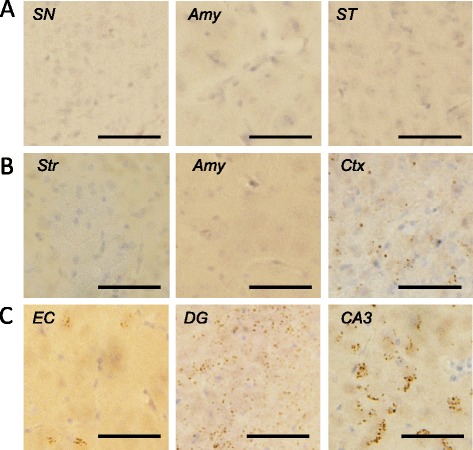
**Induction of phosphorylated TDP-43-positive structures in WT mice injected with αsyn fibrils at 1 month after injection. (A)** Phosphorylated TDP-43 was not accumulated in mice injected into SN, based on staining with anti-pS409/410 antibody. **(B)** Dot-like TDP-43 inclusions were observed in mice injected into Str. **(C)** Dot-like TDP-43 inclusions were also detected in mice injected into EC. SN: substantia nigra, Amy: amygdala, ST: stria terminalis, Str: striatum, Ctx: cortex, EC: entorhinal cortex, DG: dentate gyrus. Scale bar represents 50 μm.

**Figure 5:**
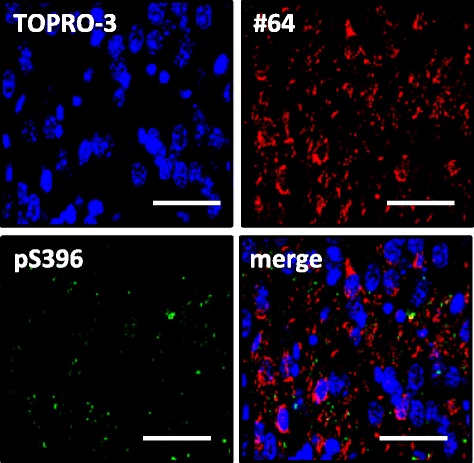
**Dot-like ptau-positive structures showed little colocalization with psyn pathology in hippocampus of mice injected with αsyn fibrils into EC at 1 month after injection.** Brain sections were double-labeled with anti-psyn antibody (#64, red) and anti-ptau antibody (pS396, green). Scale bar represents 50 μm.

To investigate whether other pathologies are also induced by the injection of αsyn fibrils, we performed IHC analysis using anti-tau, anti-TDP and anti-Aβ antibodies. No tau pathology was observed in mice injected into SN (Figure 3A). However, surprisingly, in mice injected into Str, pS396-positive dot-like structures were observed in Str, amygdala, and cortex (Figure 3B). Anti-phosphorylated tau (ptau) antibody AT8 also stained these structures in Str (Figure 3C). Similar ptau-positive dot-like structures were also observed with anti-pS396 antibody in EC, dentate gyrus and CA3 of the mice injected into EC, and were most frequent in CA3 (Figure 3D). Similar staining was observed in CA3 and dentate gyrus with AT8 antibody (Figure 3E). Furthermore, phosphorylated TDP-43 was also accumulated in mice injected into Str and EC (Figure 4B,C), although it was not detected in mice injected into SN (Figure 4A) at 1 month after injection. Aβ pathology was never observed in αsyn fibril-injected mice, regardless of injection site (Additional file 1: Figure S3). The tau and TDP-43 pathologies differed from psyn pathology in both shape and localization; most psyn pathologies were not colocalized with ptau-positive structures and the overlap was small (Figure 5).

**Figure 6:**
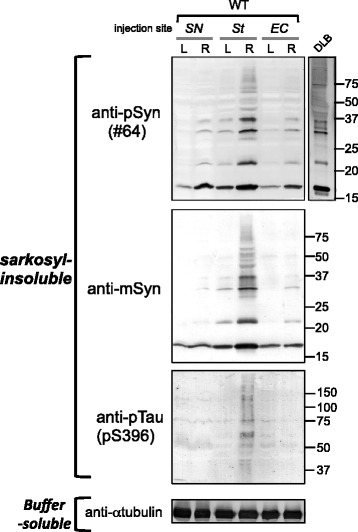
**Intracerebral injections with αsyn fibrils induced accumulation of endogenous αsyn and tau in WT mice.** At 3 months after injection, sarkosyl-insoluble fractions were prepared from the right and left brains and analyzed by immunoblotting with #64, anti-mouse syn and anti-pS396 antibodies. Band patterns of insoluble psyn were identical to that of DLB brain. Insoluble ptau was also accumulated in mouse brains showing abundant accumulation of αsyn. L: left brain, R: right brain.

To confirm the accumulation of these proteins and to analyze them biochemically, we next investigated sarkosyl-insoluble fractions of these mice brains at 3 months after injection into SN, Str or EC (Figure 6). Sarkosyl-insoluble psyn was detected in both the right and left hemispheres of all these mice, though it was more abundant on the injection side. The accumulation was most abundant on the injection side (right brain) in mice injected into Str, and less abundant on the uninjected side in mice injected into SN or EC. The banding patterns of sarkosyl-insoluble psyn were identical among these mice, regardless of the injection site, and were indistinguishable from that of DLB brain (Figure 6 upper). Anti-mouse αsyn antibody showed the same banding pattern as psyn antibody (Figure 6 middle). The 15, 22, 30 and 35 kDa bands correspond to monomer, monoubiquitinated αsyn, dimer and ubiquitinated dimer, respectively. Moreover, sarkosyl-insoluble ptau was detected in the right hemisphere of mice injected into Str, where the most abundant tau inclusions were observed (Figure 6 lower). On the other hand, αsyn and tau accumulations were not observed in αsyn KO mice injected with fibrils into Str (Additional file 1: Figure S4). These results indicate that inoculation of αsyn fibrils converted mouse αsyn at the injection sites to an abnormal form, that this change propagated from the injection site to the contralateral side of the brain, and that inoculation into Str also induced tau pathology.

**Figure 7:**
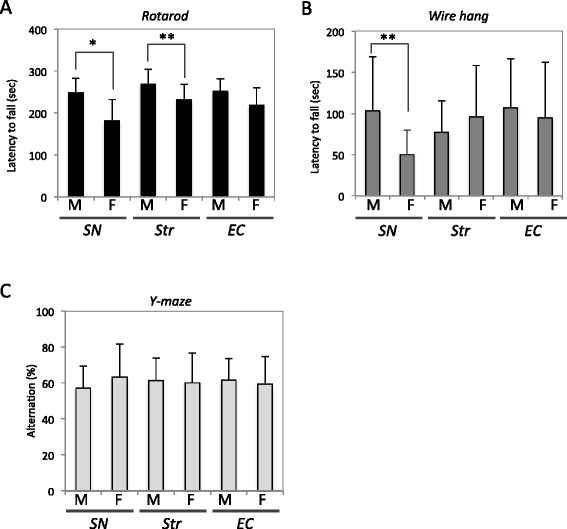
**Fibril-injected mice showed motor dysfunctions compared to monomer-injected mice at 3 months after injection. (A)** Rotarod test. Mice injected into SN and Str showed lower performance in the rotarod test. **(B)** Wire hang test. **(C)** Y-maze test. All error bars indicates mean ± S. E. M. *p < 0.01, **p < 0.05. M: αsyn monomer-injected, F: αsyn fibril-injected.

Next, we analyzed motor and cognitive functions of these mice at 3 months after injection (Figure 7). Mice injected with αsyn fibrils into SN and Str showed poorer performance on the rotarod test compared with control mice injected with soluble αsyn (Figure 7A). Mice injected into SN also performed poorly on the wire hang test (Figure 7B). Cognitive dysfunction was not observed in any group in the Y-maze test (Figure 7C).

**Figure 8:**
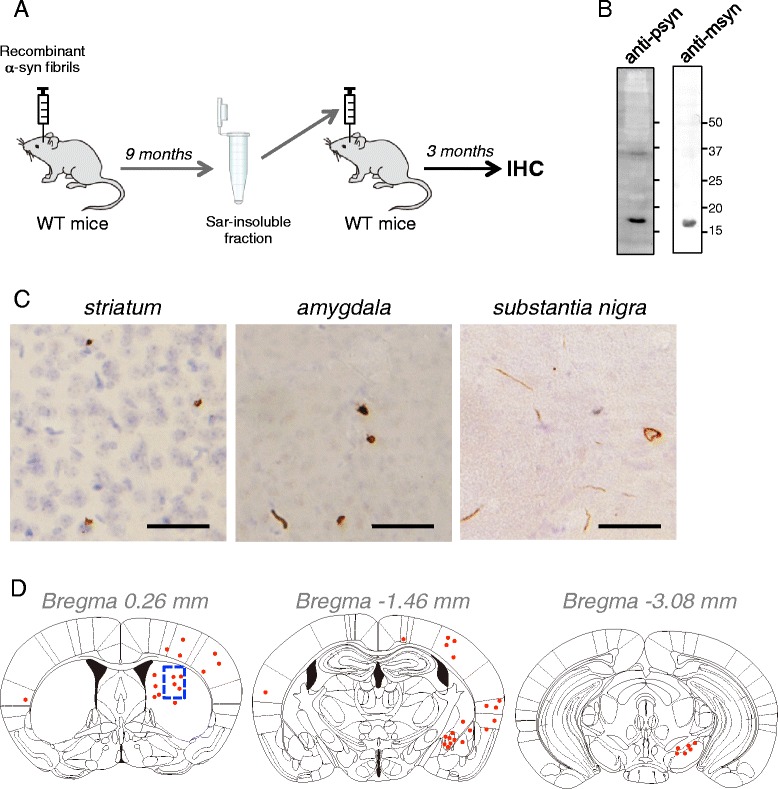
**Sequential transmission of insoluble αsyn. (A)** Schematic diagrams of transmission experiments. Sarkosyl-insoluble fractions were prepared from WT mouse brains injected with recombinant αsyn fibrils at 9 months post-injection and intracerebrally injected into 4-month-old WT mice. At 3 months after injection, transmission of αsyn pathology was analyzed by IHC. **(B)** Immunoblot of sarkosyl-insoluble fractions from WT mice at 9 months post-injection with #64 antibody and anti-msyn antibody. **(C)** Psyn pathology was observed mainly in Str, amygdala and SN with 1175 antibody. Scale bar represents 50 μm. **(D)** Distribution of psyn pathology in WT mouse brains injected with sarkosyl-insoluble fractions. Blue-dashed box and red dots indicate the injection site (Str) and psyn pathology, respectively.

Finally, to examine whether insoluble αsyn induced in WT mice shows prion-like propagation behavior, we assessed the transmissibility of insoluble αsyn prepared from fibril-injected WT mouse brains. In brief, sarkosyl-insoluble αsyn was prepared from WT mouse brains injected with recombinant αsyn fibrils and was injected into Str of other WT mouse brains (Figure 8A, B). Induction and propagation of psyn pathology were examined by IHC. At 3 months after injection, psyn pathology was observed in Str (0.26 mm anterior to bregma) and had also propagated to amygdala (1.46 mm posterior to bregma) and SN (3.08 mm posterior to bregma) (Figure 8C). The distribution of psyn pathology is illustrated in Figure 8D. These data clearly showed that insoluble αsyn derived from WT mice injected with αsyn fibrils exhibits prion-like transmissibility.

## Discussion

Luk et al. and we have established that insoluble αsyn shows prion-like propagation behavior in WT mouse brain [20,21], but the mechanism of spreading remains poorly understood. In this study, we investigated the spread and distribution pattern of psyn pathology in mouse brain injected with recombinant αsyn fibrils into three different brain regions: SN, Str, and EC. We assessed the distribution at 1 month post injection by using highly sensitive IHC. Pretreatment of brain sections with formic acid and heat enabled detection of psyn pathology at only 1 month after injection. When αsyn fibrils were injected into SN, psyn pathology only appeared in the central nucleus of amygdala and stria terminalis, which are located far from SN, while there was no detectable psyn pathology around SN (Figure 1A). Amygdala is connected with SN [30], and stria terminalis serves as a major output pathway of the central nucleus of amygdala. These findings strongly indicate that spreading of psyn pathology does not occur by simple diffusion or nonspecific transport. In the case of injection into Str, psyn pathology was observed in amygdala, SN and a wide range of cortices (Figure 1B). Str has direct projection to SN and amygdala [31], and many parts of the neocortex innervate the Str [32]. Injection into EC induced pathology in EC, dentate gyrus, hippocampal CA3, fimbria and septal nucleus (Figure 1C). Dentate gyrus receives projection from EC via the perforant pathway, and septal nucleus and fimbria have direct connections with hippocampus. Therefore, the data strongly suggest that propagation of pathological αsyn occurs via axonal transport and a trans-synaptic pathway, in accordance with reports that αsyn fibrils can be internalized by neurons and transferred from axons to second-order neurons in culture [33] and in animal models [34,35]. In patients with sporadic PD, the distribution of Lewy bodies and Lewy neurites seems to spread retrogradely [18,36]. In the present study, focusing on Str and SN, injection with αsyn fibrils into Str induced αsyn pathology in SN at 1 month after injection (Figure 1B). However, injection into SN did not induce pathology in Str at that time (Figure 1A), and pathology only became apparent in Str at 3 months after injection [21], indicating there is a dominant direction of spread. Propagation from EC to dentate gyrus via the perforant pathway might occur via anterograde transport (Figure 1C). At least under our experimental conditions, propagation of αsyn seems to occur via both anterograde and retrograde transport processes. Thus, the predominant direction of spread presumably depends on cell types or brain areas. Similarly, tau is also reported to propagate via a trans-synaptic pathway in animal models [37,38]. Thus, axonal transport and trans-synaptic transport appear to be common pathways of propagation of intracellular aggregated proteins.

In addition, we found that tau and TDP-43 accumulation also occurred in WT mice injected with αsyn fibrils into Str and EC at 1 month after injection (Figures 3B-E and 4). The morphological patterns of tau and TDP-43 accumulation were apparently different from that of αsyn pathology (Figures 2, 3 and 4) and there was little colocalization (Figure 5), as in DLB brains [39,40] and a DLB mouse model [41]. Recently, Guo et al. reported that there are two strains of recombinant αsyn fibrils, strains A and B, and the two strains differently affect tau inclusion formation [42]. They reported that strain A (preformed fibrils) only infrequently induced tau inclusions and psyn pathology showed little colocalization with tau inclusions, whereas strain B (generated through repetitively seeded fibrillization *in vitro*) efficiently induced tau inclusions that were highly colocalized in neurons. The αsyn fibrils we used in this study are similar to strain A, and in agreement with their work [42], we also detected small amounts of tau inclusions that showed little colocalization with αsyn pathology. In addition, tau accumulation was not observed in αsyn KO mice injected with αsyn fibrils by biochemical analysis (Additional file 1: Figure S4) or IHC (Additional file 1: Figure S2B). Thus, tau accumulation was induced by αsyn accumulation, and this occurred through a synergistic effect rather than a cross-seeding effect. The tau accumulation might be caused by a secondary effect of αsyn accumulation, such as dysfunction of cellular activity [43,44] or abnormality in protein degradation machinery [45].

Biochemical analysis clearly showed that accumulated αsyn was phosphorylated and ubiquitinated similarly to that in DLB brain, regardless of injection site (Figure 6). This indicates that injection with the same fibrils as seeds induces αsyn aggregation in the same fashion, as is the case with prion strains.

Furthermore, αsyn fibril-injected mice showed modest motor abnormalities compared to the monomer-injected mice at 3 months after injection (Figure 7). This strongly suggests that propagation of psyn pathology induced motor phenotypes, although we could not detect cognitive dysfunction in the Y-maze test. It is possible that this is because we injected αsyn fibrils unilaterally, and the functions of the contralateral side of brain might be well maintained. In our previous study, we could not detect any abnormalities in fibril-injected mice at 6 months after injection [21]. The discrepancy may have arisen from differences in the test conditions, because in the previous study, we used female mice injected with human αsyn fibrils, whereas in this study we used male mice injected with mouse αsyn. Mouse αsyn fibrils propagate more efficiently in WT mice than do human fibrils [21], and we think this was the main reason why we could detect motor abnormalities in the present study.

We next examined if insoluble αsyn accumulated in WT mice shows transmissibility. Our results demonstrate that insoluble αsyn accumulated in WT mice can induce αsyn pathology in other WT mice (Figure 8C, D), analogously to prion transmission. We also examined intraperitoneal injection or oral administration with αsyn fibrils into WT mice (see Materials and methods), but failed to detect any psyn pathology in the central nervous system at 6 months or 14 months after injection, respectively (data not shown).

## Conclusions

Intracerebral injection with αsyn fibrils into WT mouse brains enables to induce phosphorylated αsyn pathology and the distribution of pathology depends on the injection sites. Furthermore, αsyn pathology has a synergistic effect on tau and TDP-43 aggregation. We conclude that αsyn fibrils have prion-like transmissibility and it might spread via axonal and trans-synaptic transports in mouse brains.

## Additional file

Additional file 1: Table S1. Antibodies used in this study. **Figure S1.** HPLC charts of recombinant mouse αsyn used in this study. Mouse syn monomer showed only one peak that is derived from msyn monomer. Mouse syn fibril gave two peaks at 0.14 min (guanidine HCl) and at 6.8 min (msyn fibril). **Figure S2.** αSyn and tau accumulation was never observed in fibril-injected αsyn KO mice at 3 months after injection. **(A)** No psyn-positive pathology was observed with 1175 antibody. **(B)** Tau accumulation was not detected with pS396 antibody. Str: striatum, Amy: amygdala, SN: substantia nigra, sensory ctx: sensory cortex. Scale represents 50 μm. **Figure S3.** Aβ accumulation was not observed in αsyn fibril-injected WT mice at 1 month post injection. Sections were stained with anti-mouse Aβ antibody. Mice injected into SN **(A)**, Str **(B)**, and EC **(C)**. SN: substantia nigra, Amy: amygdala, ST: stria terminalis, Str: striatum, Ctx: cortex, EC: entorhinal cortex, DG: dentate gyrus. Scale represents 50 μm. **Figure S4.** Biochemical analysis of αsyn KO mice injected with human αsyn fibrils. The brain was divided into two parts at the longitudinal fissure of the cerebrum. Sarkosyl-insoluble fractions were obtained from the right and left brains, and analyzed by immunoblotting with #64, LB509 or anti-mouse αsyn antibodies. Exogenous human αsyn fibrils were detected in sarkosyl-insoluble fractions and were not phosphorylated at 0 and 7 days after injection. They were subsequently degraded and disappeared within 30 days post injection. Phosphorylated αsyn accumulation was never observed at 90 days after injection.
