# Supplementary material for: Pathological alpha-synuclein propagates through neural networks
Source: Acta Neuropathol Commun. 2014 Aug 6;2:88. doi: 10.1186/s40478-014-0088-8 (PMC4147188; doi:10.1186/s40478-014-0088-8)

Table S1

Antibodies used in this study.

Antibodies *(epitope)*	*Type*	*Dilution*	*Source*
**1175** (phosphorylated αsyn (pS129))	Rabbit polyclonal	1/2000	[26]
**#64** (phosphorylated αsyn (pS129))	Mouse monoclonal	1/1000	[5]
**anti-mouse syn** (mouse αsyn)	Rabbit monoclonal	1/1000	Cell Signaling Technology
**LB509** (human αsyn specific)	Mouse monoclonal	1/1000	[27]
**pS396** (phosphorlated tau (pS396))	Rabbit polyclonal	1/2000	CALBIOCHEM
**Biotin-AT8** (phosphorlated tau (pS202/pT205))	Mouse monoclonal	1/50	Thermo
**pTDP43** (phosphorylated TDP-43 (pS409/410))	Rabbit polyclonal	1/1000	[28]
**anti-Aβ** (mouse Aβ (1-24) peptide)	Rabbit polyclonal	1/1000	kindly gifted from Dr. Kametani
**α-tubulin**	Mouse monoclonal	1/3000	Sigma

Figure S1

HPLC charts of recombinant mouse αsyn used in this study. Mouse syn monomer showed only one peak that is derived from msyn monomer. Mouse syn fibril gave two peaks at 0.14 min (guanidine HCl) and at 6.8 min (msyn fibril).

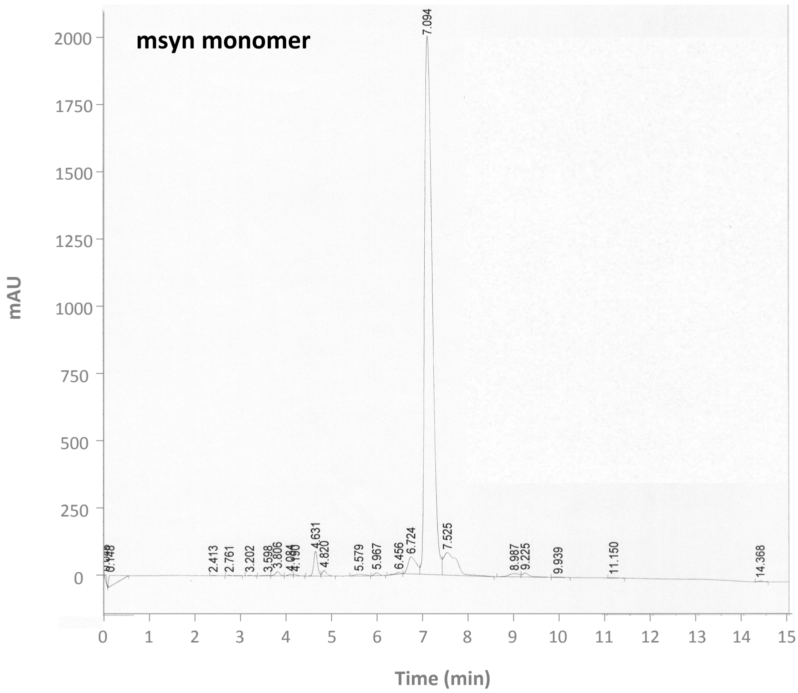

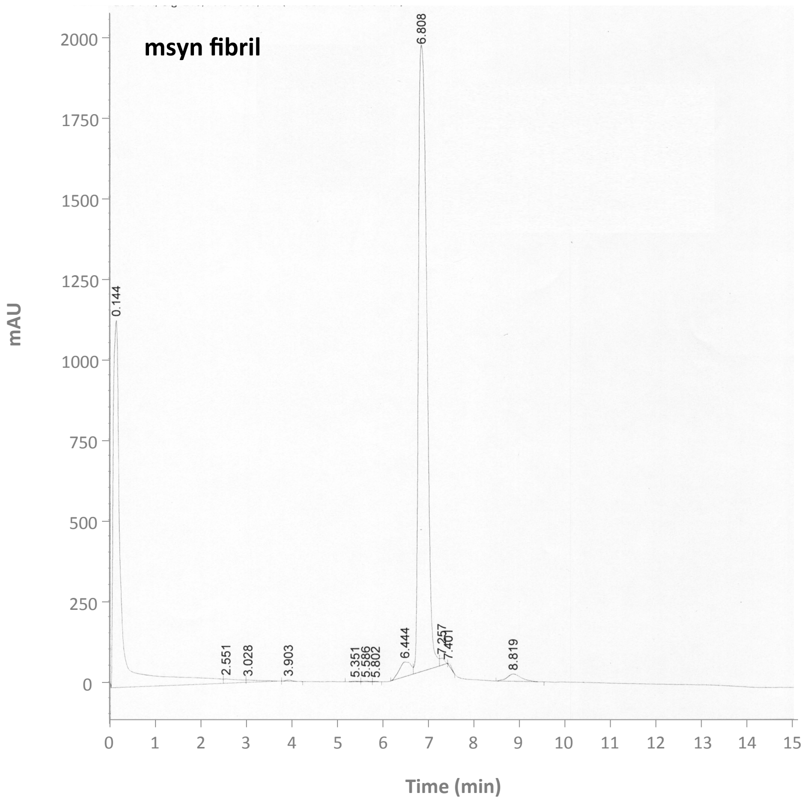

Figure S2

αSyn and tau accumulation was never observed in fibril-injected αsyn KO mice at 3 months after injection. (A) No psyn-positive pathology was observed with 1175 antibody. (B) Tau accumulation was not detected with pS396 antibody. Str: striatum, Amy: amygdala, SN: substantia nigra, sensory ctx: sensory cortex. Scale represents 50 μm.

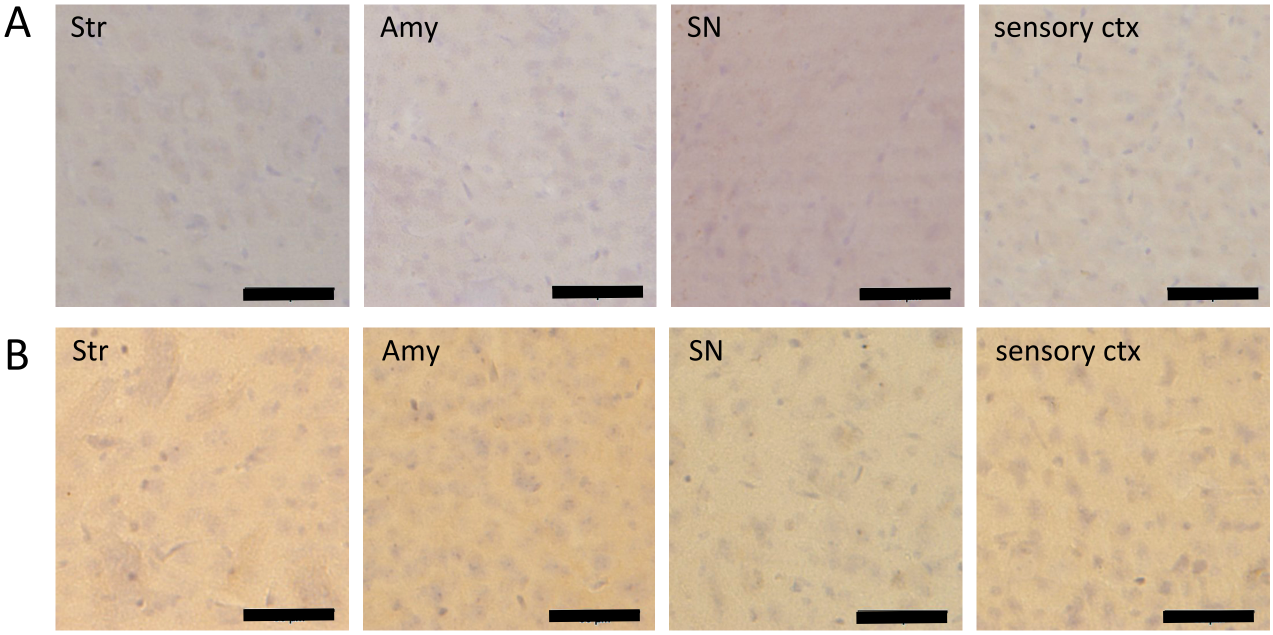

Figure S3

Aβ accumulation was not observed in αsyn fibril-injected WT mice at 1 month post injection. Sections were stained with anti-mouse Aβ antibody. Mice injected into SN (A), Str (B), and EC (C). SN: substantia nigra, Amy: amygdala, ST: stria terminalis, Str: striatum, Ctx: cortex, EC: entorhinal cortex, DG: dentate gyrus. Scale represents 50 μm.

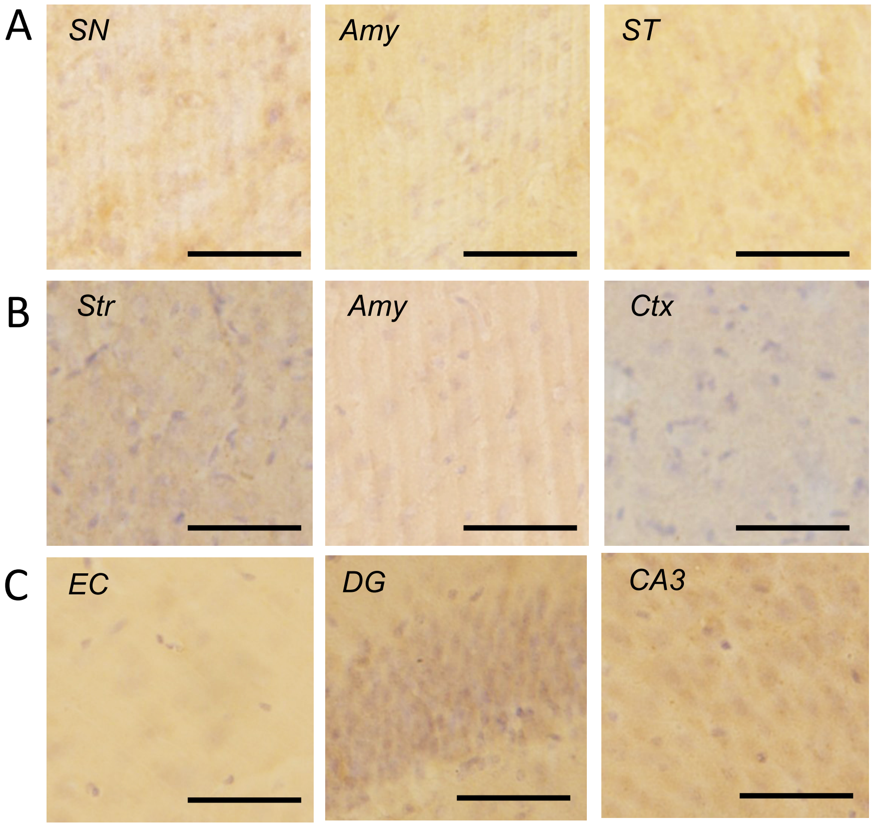

Figure S4

Biochemical analysis of αsyn KO mice injected with human αsyn fibrils. The brain was divided into two parts at the longitudinal fissure of the cerebrum. Sarkosyl-insoluble fractions were obtained from the right and left brains, and analyzed by immunoblotting with #64, LB509 or anti-mouse αsyn antibodies. Exogenous human αsyn fibrils were detected in sarkosyl-insoluble fractions and were not phosphorylated at 0 and 7 days after injection. They were subsequently degraded and disappeared within 30 days post injection. Phosphorylated αsyn accumulation was never observed at 90 days after injection.

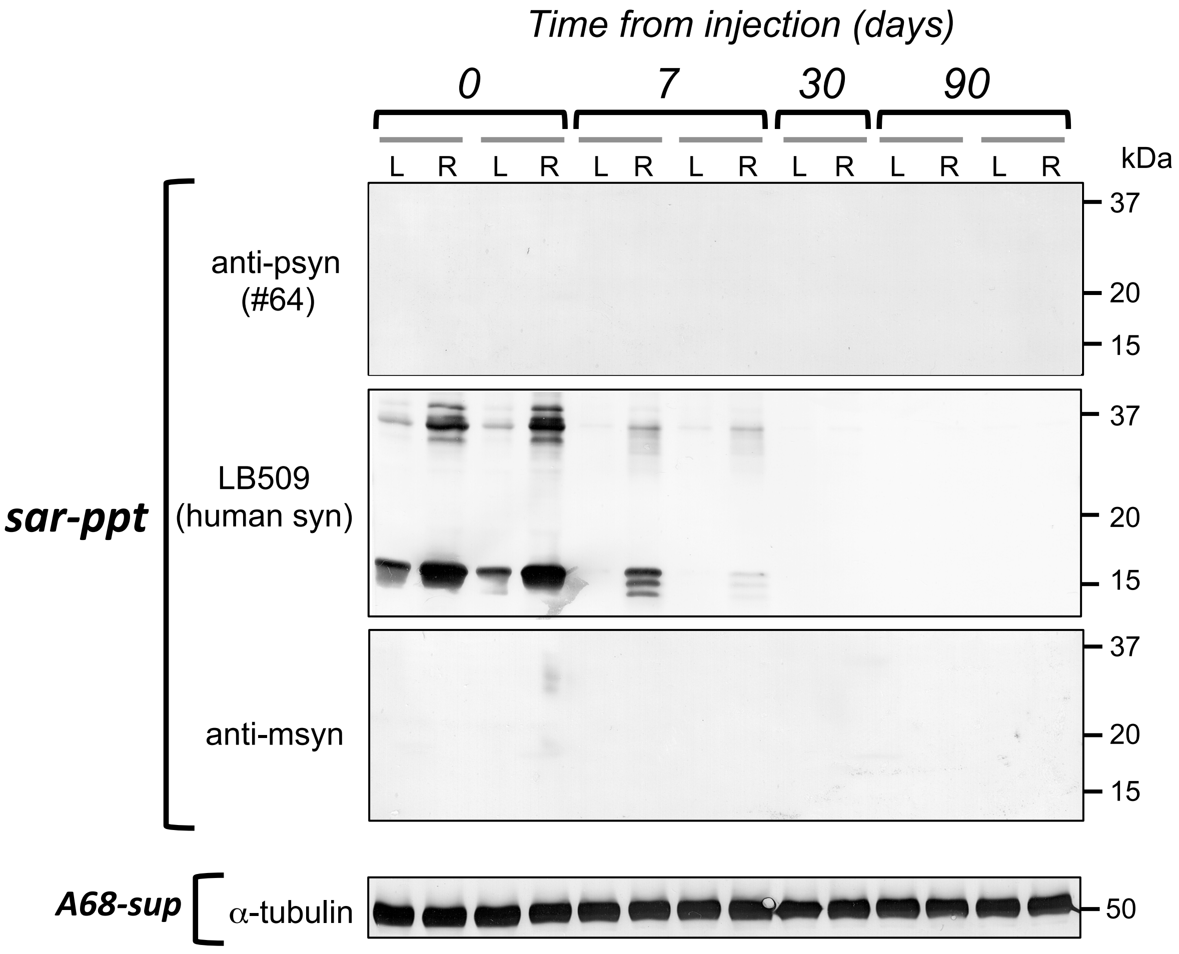

Supplement: Additional file 1: Table S1. — Antibodies used in this study. Figure S1. HPLC charts of recombinant mouse αsyn used in this study. Mouse syn monomer showed only one peak that is derived from msyn monomer. Mouse syn fibril gave two peaks at 0.14 min (guanidine HCl) and at 6.8 min (msyn fibril). Figure S2. αSyn and tau accumulation was never observed in fibril-injected αsyn KO mice at 3 months after injection. (A) No psyn-positive pathology was observed with 1175 antibody. (B) Tau accumulation was not detected with pS396 antibody. Str: striatum, Amy: amygdala, SN: substantia nigra, sensory ctx: sensory cortex. Scale represents 50 μm. Figure S3. Aβ accumulation was not observed in αsyn fibril-injected WT mice at 1 month post injection. Sections were stained with anti-mouse Aβ antibody. Mice injected into SN (A), Str (B), and EC (C). SN: substantia nigra, Amy: amygdala, ST: stria terminalis, Str: striatum, Ctx: cortex, EC: entorhinal cortex, DG: dentate gyrus. Scale represents 50 μm. Figure S4. Biochemical analysis of αsyn KO mice injected with human αsyn fibrils. The brain was divided into two parts at the longitudinal fissure of the cerebrum. Sarkosyl-insoluble fractions were obtained from the right and left brains, and analyzed by immunoblotting with #64, LB509 or anti-mouse αsyn antibodies. Exogenous human αsyn fibrils were detected in sarkosyl-insoluble fractions and were not phosphorylated at 0 and 7 days after injection. They were subsequently degraded and disappeared within 30 days post injection. Phosphorylated αsyn accumulation was never observed at 90 days after injection. [file 40478_2014_88_MOESM1_ESM.pdf]
